# Stakeholder views on the potential impact of a sugar-sweetened beverages tax on the budgets, dietary intake, and health of lower and higher socioeconomic groups in the Netherlands

**DOI:** 10.1186/s13690-020-00507-x

**Published:** 2020-11-24

**Authors:** Sanne K. Djojosoeparto, Michelle Eykelenboom, Maartje P. Poelman, Maartje M. van Stralen, Carry M. Renders, Margreet R. Olthof, Ingrid H. M. Steenhuis, Carlijn B. M. Kamphuis, on behalf of the PEN Consortium

**Affiliations:** 1 Department of Human Geography and Spatial Planning, Faculty of Geosciences, Utrecht University, Utrecht, the Netherlands; 2 Department of Health Sciences, Faculty of Science, Vrije Universiteit Amsterdam, and Amsterdam Public Health Research Institute, Amsterdam, the Netherlands; 3 Chair group Consumption and Healthy Lifestyles, Department of Social Sciences, Wageningen University & Research, Wageningen, the Netherlands; 4 Department of Interdisciplinary Social Science, Faculty of Social and Behavioural Sciences, Utrecht University, Utrecht, the Netherlands

**Keywords:** Stakeholder views, Sugar-sweetened beverages tax, Budgets, Dietary intake, Health, Socioeconomic groups, Inequalities

## Abstract

**Background:**

Socioeconomic inequalities in overweight and obesity exist in many European countries. A sugar-sweetened beverages (SSB) tax may contribute to a reduction of these inequalities. However, in the Netherlands, the government decided to not (yet) introduce an SSB tax, although the government has acknowledged its potential to be pro-equity. Understanding how various stakeholder groups perceive the potential effects of an SSB tax on different socioeconomic groups may provide useful insights into equity-related considerations in the debate whether or not to implement an SSB tax. This study aims to gain insight into the perceptions of stakeholder groups in the Netherlands on (1) the effects of an SSB tax on the budgets of lower and higher socioeconomic groups and (2) the impact of an SSB tax on socioeconomic inequalities in dietary intake and health.

**Methods:**

Semi-structured interviews were conducted in 2019 with 27 participants from various stakeholder groups in the Netherlands (i.e. health and consumer organizations, health professional associations, trade associations, academia, advisory bodies, ministries and parliamentary parties). Data were analyzed using a thematic content approach.

**Results:**

Participants from all stakeholder groups indicated that an SSB tax would have a larger impact on the budgets of lower socioeconomic groups. Participants from nearly all stakeholder groups (except trade associations) mentioned that an SSB tax could have greater health benefits among lower socioeconomic groups as these often have a higher SSB consumption and are more likely to be overweight or obese. Some participants mentioned that an SSB tax may have no or adverse health effects among lower socioeconomic groups (e.g. compensation of lower SSB consumption with other unhealthy behaviours). Some participants emphasised that an SSB tax should only be introduced when accompanied by other interventions (e.g. offering healthy alternatives), to make it easier for lower socioeconomic groups to lower their SSB consumption in response to an SSB tax, and to prevent adverse health effects.

**Conclusions:**

Participants believed an SSB tax could contribute to a reduction in socioeconomic inequalities in dietary intake and health. However, additional interventions facilitating the reduction of SSB consumption in lower socioeconomic groups were recommended.

## Background

According to the World Health Organization (WHO), obesity has nearly tripled since 1975 worldwide [1]. In 2016, more than 1.9 billion adults (39% of the global population) were overweight, over 650 million of whom (13% of the global population) were obese [1]. In the WHO European Region, 58% of the adult population was overweight in 2014 [2]. People with a lower educational level are more likely to be overweight and obese than those with a higher educational level in most European countries [3], and a widening of absolute socioeconomic inequalities in obesity prevalence has been observed in 15 European countries [4].

Unhealthy diets are a leading risk factor for overweight and obesity, as well as for other diet-related non-communicable diseases, such as cardiovascular diseases, type 2 diabetes, musculoskeletal disorders (especially osteoarthritis) and some cancers [1, 2, 5]. Unhealthy diets are characterized by excessive intake of saturated fats, trans fats, sugar, and salt, largely due to increased consumption of highly processed, energy-dense manufactured foods, like sugar-sweetened beverages (SSBs). Previous studies have shown that unhealthy diets are more common among people with a lower socioeconomic position (SEP) [6], and that lower socioeconomic groups consume more SSBs than higher socioeconomic groups [7–10]. A study of 11-year old children revealed that children with a low SEP consumed 0,63 l of SSBs more per week than children with a high SEP. [10]

The WHO Commission on Ending Childhood Obesity noted that fiscal policies may encourage consumers – and especially those on low income - to make healthier choices [11]. The WHO sees taxation of SSBs as the most feasible fiscal policy tool to implement on a large scale. SSBs are an easy-to-define category of products that are dense in energy and poor in nutrients, which have healthier and less expensive substitutes (e.g. water) [12]. Furthermore, SSBs are a major driver of increased weight gain and type 2 diabetes which provides a rationale for government action [13, 14].

As lower socioeconomic groups are more sensitive to price increases [15] and consume more SSBs than higher socioeconomic groups, an SSB tax may have larger positive effects on the healthfulness of diets of lower socioeconomic groups [12, 15]. Evidence from countries in which an SSB tax already has been implemented shows a decrease in SSB consumption [13, 16, 17], with a greater reduction among those with a lower SEP. [8] As a result, an SSB tax, as a component of a comprehensive approach, could contribute to reduced dietary inequalities, and ultimately, improved health equity [6, 12, 18, 19].

Although an increasing number of countries worldwide and in Europe have introduced an SSB tax [20], the Netherlands has not (yet) implemented such a tax. In the Netherlands a value-added tax (VAT) rate of 9% is applied to all food and beverages [21] and an additional consumption tax of 8,83 eurocent per liter is applied to non-alcoholic drinks (i.e. fruit and vegetable juices, soft drinks and mineral water), with no distinction made between SSBs and sugar-free beverages [22]. In 2020, the Dutch National Institute for Public Health and the Environment (RIVM), commissioned by the Ministry of Health, compared the characteristics and effects of an SSB tax in the United Kingdom, France and Norway [23]. Results from this study showed a reduction in SSB purchases and an increase in purchases of healthy alternatives after implementation of an SSB tax. However, the RIVM notes that it is unclear to what extent these changes in purchases are a direct consequence of the SSB taxes. In addition, the study showed that there is evidence from the United Kingdom that an SSB tax may encourage manufacturers to reduce sugar levels in soft drinks.

In a written response to the RIVM study, the Dutch government recognized the potential of an SSB tax (in addition to the efforts made by the industry) to reduce sugar consumption and its potential to stimulate product reformulation [24]. The government specifically mentioned that an SSB tax could reduce sugar consumption among people with a lower SEP as they will be more sensitive to price increases. However, the government has decided to not (yet) introduce an SSB tax and refers to the agreement with the food industry on self-regulated measures to reduce sugar levels in soft drinks. These self-regulated measures have been included in the ‘National Prevention Agreement’, an agreement of the Dutch government with more than seventy public and private organizations to reduce the prevalence of overweight and obesity, smoking and alcohol consumption [25].

Thus, although evidence suggests an SSB tax may have the potential to reduce SSB consumption and be pro-equity, this does not imply that stakeholders involved in the debate whether or not to implement an SSB tax are sensitive to this argument. The views of ministers and other governmental officials are likely influenced by the opinions and lobbying of other stakeholder groups in the SSB-tax-debate, which could slow down or even block the decision-making process [26, 27]. Various studies reported concerns among stakeholders about the regressive effect of an SSB tax; the tax would have a larger impact on the (often) smaller budgets of lower socioeconomic groups, which is considered unfair [28].

It is important to understand how different stakeholder groups (e.g. health professional associations, academics, policy makers) perceive the equity effects of an SSB tax, and which different equity-related arguments may influence the decision-making process of an SSB tax. These perspectives would lead to a greater understanding on how different stakeholders believe an SSB tax could contribute to socioeconomic inequalities in dietary intake and health and may address issues where contradictory views exist. Furthermore, a better understanding of the views of different stakeholder groups may also provide useful information on how the pro-equity effect of an SSB tax could be increased.

These insights are especially relevant for governments considering to implement an SSB tax, given the persistence of health inequalities in many European countries. This study therefore aims to gain insight into the perceptions of different stakeholder groups in the Netherlands on (1) the differential effects of an SSB tax on the budgets of lower and higher socioeconomic groups and (2) the impact of an SSB tax on socioeconomic inequalities in dietary intake and health.

## Methods

### Study design and participant recruitment

Semi-structured interviews were conducted with Dutch stakeholders from various professional backgrounds and a wide range of sectors who have an interest in the potential implementation of an SSB tax in the Netherlands: ministries and parliamentary parties, advisory bodies, academia, trade associations, health professional associations, health and consumer organizations (Table 1). These stakeholder groups, their role and their potential interest in the SSB tax decision-making process are further described below.

**Table 1:** Study participants

Stakeholder Group	Participants approached (*n* = 46)	Participants declined (*n* = 19)	Participants included (*n* = 27)
Parliamentary parties	10	No response (*n* = 2) No time (*n* = 1) No reason (*n* = 4)	Members of the Dutch parliament/ politicians (*n* = 3)
Ministries	8	No response (*n* = 2) Insufficient knowledge on taxation of SSBs according to the stakeholder (*n* = 1) No reason (*n* = 2)	Policymakers from various ministries (*n* = 3)
Advisory bodies	4	No response (*n* = 1) Insufficient knowledge on taxation of SSBs according to the stakeholder (*n* = 1)	Representatives of a governmental, non-profit advisory body (*n* = 2)
Academia	10	No time (=1)	Academics in the field of obesity prevention, nutrition and health, preventive dentistry, behavioural science, health economics, tax law, political science, medical ethics or social epidemiology (*n* = 9)
Trade associations	5	No response (*n* = 1)	Representatives of trade associations for food and beverages manufacturers, hospitality businesses or the catering industry (*n* = 4)
Health professional associations	3	NA	Representatives of non-governmental, non-profit professional associations of physicians or dentists (*n* = 3)
Health and consumer organizations	6	No response (*n* = 2) Insufficient knowledge on taxation of SSBs according to the stakeholder (*n* = 1)	Representatives of governmental or non-governmental, non-profit consumer organizations and health organizations in the field of nutrition and health promotion (*n* = 3)

*The Dutch Ministry of Health, Welfare and Sport* develops national policies to reduce the prevalence of overweight and obesity among children and adults in the Netherlands, which could include an SSB tax. However, an SSB tax is not included in the ‘National Prevention Agreement’, a package of measures to reduce the prevalence of overweight and obesity [25]. *The Dutch Ministry of Finance* has an important role in adjusting the tax regulations when the decision to implement an SSB tax has been taken and is responsible for the implementation of the SSB tax [29].

*Parliamentary parties* have three key responsibilities: 1) to propose, review and pass laws in collaboration with the government, 2) to review the Cabinet’s (i.e. the Prime Minister, the other ministers and the State Secretaries) implementation of legislation and all other government actions and 3) to represent the Dutch voters [30]. In the Netherlands various parliamentary parties exist (from left-wing to right-wing parties). These parties participate in the House of Representatives (Second Chamber), elected directly by Dutch citizens and the Senate (First Chamber), chosen by the members of the State-Provincial.

*Advisory bodies* (representatives of a governmental, non-profit advisory body) conduct health-related research and monitoring studies commissioned by the government [31, 32]. They provide the government with independent advice on how to promote health and safeguard a healthy environment.

*Academia* have expertise in certain fields, such as obesity prevention and nutrition and health. Academia conduct independent scientific research on for example the effects or acceptability of an SSB tax. These results can be used in the development of policies [32]. Academia were also involved in the development and negotiations of the National Prevention Agreement [25].

*Trade associations* (representatives of food and beverages manufacturers, hospitality businesses or the catering industry) influence government decisions such as the implementation of an SSB tax through their lobby activities [28]. They are involved in the development and negotiations of several government policies, such as the National Prevention Agreement [25] and the Agreement on Product Improvement which has been signed in 2014 [33]. When the decision to implement an SSB tax has been taken, food and beverages manufacturers, hospitality businesses and the catering industry have to implement the tax.

*Health professional associations* (representatives of non-governmental, non-profit professional associations of physicians or dentists) and *health and consumer organizations* (representatives of governmental or non-governmental, non-profit consumer organizations and health organizations in the field of nutrition and health promotion) may influence the decision-making process of implementing an SSB tax by lobbying to the government and promoting the importance of healthy dietary lifestyles to prevent diseases [32, 34]. They are also involved in diverse platforms of interaction with the government and many of these organizations were involved in the development and negotiations of the National Prevention Agreement [25].

To recruit participants, we used purposive sampling combined with snowball sampling, asking each participant if certain stakeholders or stakeholder groups were lacking from the initial anonymized list of stakeholders developed by the research team. We approached a total of 46 stakeholders, of whom 11 declined to participate and 8 did not return phone calls or reply to reminder e-mails (see Table 1 for reasons for non-participation). A total of 25 semi-structured interviews were held with 27 participants. One interview was held with two participants that represented two trade associations and one interview was held with two participants that represented one advisory body.

### Interview procedure

Data were collected in March, April and May 2019. Interviews were held in person, by (one of the) two members of the research team (ME and SD). Most interviews were held at the participants workplace. Three interviews were held at a neutral and for the participant easy accessible location and one interview was held at the interviewers’ workplace, at the request of the participant. The interviews were held in Dutch, using a semi-structured interview guide, with questions developed based on topics as identified in the literature [28, 35, 36]. The interviewers provided the participants with a definition of an SSB tax: a tax of at least 20% on regular soft drinks, fruit juices with added sugars, sport drinks, energy drinks and flavoured water with added sugars. The interview guide included questions about barriers and facilitators to the potential future introduction of an SSB tax in the Netherlands and advantages and disadvantages of an SSB tax, including questions about potential differential effects of an SSB tax on different socioeconomic groups. This manuscript focuses on the latter: stakeholder views on differential effects of an SSB tax on the budgets, dietary intake, and health of lower and higher socioeconomic groups. Results about perceived advantages and disadvantages of an SSB tax and barriers and facilitators to its potential introduction have been described elsewhere (Eykelenboom et al., submitted).

The interviews lasted 25 to 90 min, were audio-recorded, and transcribed verbatim by two researchers (ME and SD). A summary of the interview was sent to every participant to check for accuracy. Anonymity of the participants was assured by using identification numbers instead of names.

### Data analysis

Full interview transcripts were analysed; that is including the part on advantages and disadvantages of an SSB tax, since equity aspects were mentioned here by participants. Two researchers (ME and SD) analysed the interview transcripts with MAXQDA Qualitative Data Analysis Software (version 2018.2), using a thematic content approach [37]. ME and SD coded the first four interview transcripts of interviews with participants from four different stakeholder groups inductively line-by-line and independent of each other. Subsequently, ME and SD discussed the emergent subthemes until consensus was reached. These subthemes were then aggregated into overarching themes, which led to the development of an initial thematic map which was used for coding the next interview transcripts by either ME or SD. During the coding, ME and SD discussed the subthemes and overarching themes repetitively with each other to review themes for coherence, refine existing themes, identify and add new themes, and recode some data extracts.

All interview fragments that were coded with either ‘*health inequalities*’ or ‘*budgetary inequalities’* were further analysed for this manuscript. Fragments in which participants talked about specific views/topics in relation to these two themes have been copied from the transcripts and pasted into a separate document, including the ID-number of the participant who brought this up. The researchers checked which stakeholder group the participant belonged to. This document was used to create Table 2, which shows the specific views and topics that have been mentioned. If one or more participants from a certain stakeholder group mentioned a specific topic, this has been indicated with an ‘X’ in Table 2. All quotes in this manuscript have been translated from Dutch into English.

**Table 2:** Views on the effects of an SSB tax on higher and lower socioeconomic groups

	Stakeholder groups						
	Health and Consumer organizations	Health professional associations	Advisory bodies	Academia	Trade associa-tions	Minis-tries	Parliamentary parties
**The differential effects of an SSB tax on the budgets of lower and higher socioeconomic groups**							
An SSB tax is financially regressive	*X*	*X*	*X*	*X*	*X*	*X*	*X*
People with a lower socioeconomic position spend a bigger proportion of their income on food purchases				*X*		*X*	
People with a lower socioeconomic position consume more unhealthy foods/drinks like SSBs		*X*	*X*	*X*		*X*	*X*
People of lower socioeconomic groups cannot easily change their routines				*X*			
The regressive effect of an SSB tax is likely very marginal						*X*	
The regressive effect of an SSB tax does not need to occur when people eat healthy and consequently do not buy SSBs							*X*
**The impact of an SSB tax on socioeconomic inequalities in dietary intake and health**							
An SSB tax is progressive in terms of health benefits	*X*	*X*	*X*	*X*		*X*	*X*
People with a lower socioeconomic position have more health problems (overweight, non-communicable diseases)	*X*	*X*		*X*		*X*	*X*
People with a lower socioeconomic position consume more unhealthy foods/drinks like SSBs		*X*	*X*	*X*		*X*	*X*
An SSB tax could lead to alternative, healthier choices of lower socioeconomic groups	*X*			*X*			*X*
An SSB tax could be especially effective for the health of people with a lower SEP who are more difficult to reach by other interventions				*X*			
People of lower socioeconomic groups cannot easily change their routines				*X*			
An SSB tax could lead to compensation with other unhealthy behaviour	*X*	*X*					
An SSB tax could lead to a widening of socioeconomic inequalities in dietary intake and health		*X*		*X*			

X denotes: one or more participants in this stakeholder group mentioned the specific topic in an interview

## Results

Table 2 gives an overview of the views of different stakeholder groups on how an SSB tax would affect the budgets of lower and higher socioeconomic groups differently, and impact on inequalities in dietary intake and health. Below we elaborate on these different views.

### Differential effects of an SSB tax on the budgets of lower and higher socioeconomic groups

Participants from all stakeholder groups mentioned that an SSB tax will have a financially regressive effect. Participants (from academia and the ministries) reasoned that people with a lower SEP often have a lower income and spend a larger proportion of their income on food purchases. Consequently, an SSB tax will have a larger effect on the budgets of people with a lower SEP. Furthermore, participants (from health professional associations, advisory bodies, academia, a ministry, and parliamentary parties) reasoned that people with a lower SEP in general consume more unhealthy foods and drinks like SSBs than people with a higher SEP, which could further increase the effect of an SSB tax on their financial situation if they would maintain their usual levels of SSB consumption. There could be a chance that people with a lower SEP will stick to their usual SSB consumption levels, because they can be less capable of and/or willing to change their routines regarding consuming SSBs than people with a higher SEP, as was mentioned by two academics. An academic noted ‘ *… it is financially unbearable for people when they are not capable to make alternative choices. That is a potential problem*.’ Another academic reasoned that when an SSB tax will disproportionally affect the financial situation of people with a lower SEP, this could thus lead to a widening of budgetary inequalities.

However, a few participants, although acknowledging the regressive effect of an SSB tax, were less concerned about possible adverse financial effects. One policy maker referred to evidence which showed that the regressive effect is likely to be marginal for this specific tax, because the absolute price increase is quite low for these relatively inexpensive drinks. A politician mentioned ‘*if the [SSB] tax increases [the price of SSBs], it works regressive. And that is of course … not the intention. On the other hand, there is the discussion about if healthy eating is more expensive … and that is not the case. Thus … if you eat according to the Dutch Wheel of Five [dietary guidelines]* [38] *(and then you do not buy cola and chips because they are not included) … you can eat healthy for 5 or 6 euros per day per person. Yes, that includes less [treats] and less cookies …*. *but those are also the products you do not need*.’

### The contribution of an SSB tax to socioeconomic inequalities in dietary intake and health

Participants from all stakeholder groups, except those from trade associations, mentioned the progressive health benefits of an SSB tax. They see it as an advantage rather than a disadvantage that an SSB tax will probably disproportionally affect the budgets and SSB consumption of people with a lower SEP.

Participants (from health professional associations, advisory bodies, academia, a ministry, and parliamentary parties) mentioned that people with a lower SEP often have unhealthier dietary behaviour, including a higher consumption of SSBs. According to a few participants (from academia) unhealthier lifestyles among people with a lower SEP may be caused by, for example, less knowledge about healthy behaviour, and by being more vulnerable to *and* more exposed to an environment that stimulates unhealthy behaviour. Consequently, the prevalence of overweight and obesity is higher among people with a lower SEP and they have a greater risk of experiencing health problems (e.g. type 2 diabetes, cardiovascular disease) than people with a higher SEP according to participants (from health and consumer organizations, a health professional association, academia, a ministry, and a parliamentary party). Participants (from health and consumer organizations, academia, and a parliamentary party) reasoned that an SSB tax will increase the price of SSBs and will therefore stimulate people, especially people with a lower SEP, to buy and consume fewer SSBs and make more alternative, healthier choices, which will thus positively contribute to their health. A politician noted ‘*from a lot of research, it appears that people with a lower socioeconomic position … … live unhealthier lives. Thus, if … people with less money to spend purchase and consume more inexpensive drinks which also contain less sugar, that is, I think what we would want*.’

Academics mentioned that an SSB tax could be especially effective for the health of people with a lower SEP, who are more difficult to reach by other interventions. One of these academics noted ‘*The whole environment we are living in promotes unhealthy behaviour ….for which [lower socioeconomic] groups are more vulnerable. If they would like to improve the health of all people, and especially of these [lower socioeconomic] groups, this [an SSB tax] is an intervention that could be potentially effective. More than an intervention that depends on individual behavioural change or motivation.’*

Thus, according to a number of participants, an SSB tax stimulating people to buy fewer SSBs will contribute positively to the health of people in general, but even more to the health of people with a lower SEP. Consequently, SSB taxation may contribute to a decrease in socioeconomic inequalities in health, like a participant from an advisory body noted ‘*prevention aims to decrease socioeconomic health inequalities … this is an advantage [of an SSB tax], as this is just what it does’.* A participant from a health professional association mentioned ‘*unhealthy [foods] should be made unattractive for everybody. And the rich people will continue buying it, I can believe that. However, especially in the lower socioeconomic groups, that [unhealthy eating] is a big problem in the Netherlands. …*. *In the lower socioeconomic groups differences will be made and that is I think what is needed.*’

In contrast to the arguments above, some participants mentioned an SSB tax could also have no or adverse health effects. As was also noted in the paragraph about the financial effects of an SSB tax, two academics mentioned that people with a lower SEP can be less capable of and/or willing to change their routines regarding consuming SSBs than those with a higher SEP. One of these academics argued that ‘*if people are stuck in certain behavioural routines, they can’t adjust these very easily’. And then the point is, how strong is the incentive? Is the incentive strong enough to break through a habit?*’ These academics also mentioned that some of the additional barriers to reducing SSB consumption that may be encountered by people with a lower SEP include a fear of negatively impacting their children’s happiness or the feeling that drinking less SSBs would lower their quality of life. As an academic mentioned: ‘*A cola is just one of the last things they [people with a lower SEP] could award themselves with*’. *Thus, [the implementation of an SSB tax] is not contributing to their quality of life, because it is a larger proportion of their income and it could increase the socioeconomic differences’*.

A possible adverse effect of an SSB tax that was raised by a few participants is the possibility that a reduced SSB consumption will be compensated by an increased consumption of other unhealthy products. One participant from a health professional association mentioned: ‘*People who cannot afford it [their usual level of SSB consumption] will maybe start doing [other] things which are ‘bad’ [for their health]*. One participant from a health/consumer organization mentioned that ‘*stop drinking SSBs is, I think, for everybody a very good step for health, but on the other hand, if you compensate that with other unhealthy behaviour, that is of course not desirable*’.

If lower socioeconomic groups do not decrease their SSB consumption, or compensate a reduced SSB consumption with eating other unhealthy treats (e.g. more sweets or chocolate bars), and higher socioeconomic groups do reduce their SSB consumption in response to an SSB tax, two participants (an academic and a professional affiliated with a health professional association) reasoned this could lead to a widening of socioeconomic inequalities in dietary intake and health.

### The implementation of an SSB tax should be accompanied by other interventions targeting lower socioeconomic groups

To make it easier for lower socioeconomic groups to lower their SSB consumption in response to an SSB tax, and to prevent adverse effects, some participants emphasized that the implementation of an SSB tax should be accompanied by other interventions. According to a participant from a health/consumer organization, informing people, creating support for an SSB tax, and offering healthier alternatives that are affordable would be essential conditions for its success. One academic mentioned further: ‘ … *if you don’t ensure at the same time a decrease in prices of healthy products, then you are disproportionally affecting [lower socioeconomic] groups, because they cannot even afford themselves ….a cola. Thus, you have to make sure there are attractive [healthy] alternatives […]* *which are festive and cheerful* ’. Another academic mentioned some form of compensation for people with a lower SEP as an additional measure accompanying the implementation of an SSB tax. The academic added that this could be done by investing the revenues of the SSB tax in a fund from which supplementary interventions that benefit the health of people with a lower SEP can be financed.

## Discussion

In this study, we aimed to gain insight into the perceptions of different stakeholder groups in the Netherlands on (1) the differential effects of an SSB tax on the budgets of lower and higher socioeconomic groups and (2) the impact of an SSB tax on socioeconomic inequalities in dietary intake and health.

Participants, from all stakeholder groups, mentioned that an SSB tax would have a regressive financial effect, i.e. a larger impact on the budgets of lower socioeconomic groups. As a result of cutting down on SSBs, participants, from all stakeholder groups except trade associations, indicated an SSB tax could have greater health benefits among lower socioeconomic groups, who often have unhealthier diets (including a higher SSB consumption) and are more likely to be overweight or obese. Some participants (from academia, a health/consumer organization, and a health professional association) indicated that an SSB tax could have no effect or adverse health effects for lower socioeconomic groups (e.g. compensation of lower SSB consumption with other unhealthy behaviours). Some participants (from academia and a health/consumer organization) emphasized that an SSB tax should only be introduced when accompanied by other interventions, to make it easier for lower socioeconomic groups to lower their SSB consumption in response to an SSB tax, and to prevent adverse health effects. Examples of these interventions are educational efforts, offering healthy alternatives, decreasing the prices of healthy products, and investing the revenue of the tax in favour of people with a lower SEP.

The regressive financial effect as perceived by participants from all stakeholder groups in our study is in line with the literature. The regressive burden of taxes on food and beverage products was highlighted in 27 studies of a systematic review of empirical studies on health taxes [39]. In another systematic review on the impact of an SSB tax according to SEP [8], five studies also reported that an SSB tax is consistently financially regressive but, like a policy maker in our study noted, only to a small degree because the absolute price increase is quite low. A politician in our study mentioned that this regressive effect does not need to occur when people eat healthy according to the Dutch dietary guidelines and consequently do not buy SSBs. However, a study in the Netherlands revealed energy density was inversely related with energy costs, implying that healthier diets cost more [40].

The progressive health effect of an SSB tax as perceived by participants in our study is also corresponding with the literature. Seven modelling studies in a review on the impact of an SSB tax according to SEP [8] reported either similar reductions in body weight across socioeconomic groups or greater reductions for lower socioeconomic groups. Another systematic review of empirical studies on health taxes [39] included 15 studies which found that public health impacts are likely to be the largest for lower socioeconomic groups. In contrast, two studies in this review did not find significant differences in health impacts between socioeconomic groups. Therefore, the authors concluded that taxes on unhealthy food and beverages may contribute to a reduction in socioeconomic health inequalities, but that more research is needed.

Several authors in the literature have addressed the question whether the regressive burden of an SSB tax would be exceeded by the benefits of an SSB tax in terms of health [39, 41–44]. The outcome of this question depends on the behaviour of consumers: if people with a lower SEP substantially reduce their SSB consumption in response to an SSB tax, then the health benefits compared to the financial burden are relatively larger, making the tax less regressive. On the other hand, if a tax does *not* lead to a considerable reduction of SSB consumption among people with a lower SEP, then the benefits compared to the financial burden are relatively small [43, 44]. The review of empirical studies by Wright et al. [39] states that available research does not sufficiently address the question whether the overall benefits of food and beverage taxes (i.e. reducing consumption of unhealthy products) exceed the financial burden for people who do not reduce their consumption.

Regarding the possibility that an SSB tax has no effect, participants in our study noted that people may not be able/willing to change their SSB consumption patterns. A participant in our study emphasized that the incentive has to be strong to make people change their SSB consumption routines, especially among people with a lower SEP. This corresponds with results in the systematic review of Wright et al. [39], which states that lower and incremental taxes are less likely to achieve behaviour changes and that evidence shows a tax of at least 20% is needed to reduce the consumption of unhealthy products [39]. Participants in our study also generally recommended to implement a tax of at least 20% on all beverages with sugar (Eykelenboom et al., submitted).

A possible adverse health effect of an SSB tax noted by participants in our study is the substitution between SSBs and other unhealthy food and beverages, which is mentioned in the literature as well [39, 43, 45–49]. Various authors have highlighted the difficulties in monitoring these behavioural responses [43, 45], but we have also found some studies which examined these substitution patterns. Studies in the United States showed that the reduction in soda consumption is completely offset by increases in consumption of other high calorie drinks and therefore did not lead to significant reductions in people’s body weight [39, 46, 47]. However, Fletcher et.al [47] also mentioned that this substitution does not necessarily have to be negative in terms of health outcomes, when people substitute the SSBs for example with whole milk (which is also high in calories, but more nutritious and does not contain added sugar). Thus, even if taxation does not lower obesity rates, SSB taxes could combat poor health outcomes (e.g. type 2 diabetes, cardiovascular risk, poor dental health) associated with high levels of added sugar consumption. Studies using simulation models showed that an SSB tax could result in a decrease in energy (calories) purchases [48] and consumption [49] but would not result in substitution to sugary foods [48]. One study showed that the predicted decline in calorie intake was larger for lower socioeconomic groups than for higher socioeconomic groups [49]. In contrast, this study found that an increase in SSB prices (half-cent per ounce) would lead to an increase in sodium and fat intake as a result of product substitution [49]. Given the few and diverse studies that examined SSB substitution patterns and the difficulty of monitoring these patterns, more empirical research is needed to be able to substantiate the substitution patterns described by the participants in our study.

Participants in our study indicated that when an SSB tax has no effect or adverse health effects (i.e. people not being able to change their SSB consumption routines and/or substituting SSBs for other unhealthy foods and beverages) this could lead to an increase in health inequalities. However, evidence showing that an SSB tax increases health inequalities is scarce. A study in Chile showed a greater decline in consumption of SSBs among people with a higher SEP compared to people with a lower SEP after the introduction of a tax. This may suggest a signalling pathway, where public health messaging discourages SSB consumption, may work more effectively for high socioeconomic groups than low socioeconomic groups [16]. Such a signalling pathway could thus lead to a widening of health inequalities. The systematic review of empirical studies by Wright et.al [39]. included two studies which did not find significant differences in public health impacts between socioeconomic groups, but this would not cause an increase in health inequalities (nor a decrease in health inequalities). Considering the few studies and diverse outcomes, more studies are needed to generate evidence about the contribution of an SSB tax to health inequalities.

To simultaneously increase the effect of an SSB tax on the health of people with a lower SEP and prevent an SSB tax from increasing socioeconomic inequalities in dietary intake and health, some stakeholders in our study suggested to implement additional interventions accompanying the SSB tax. Implementing an SSB tax as part of a comprehensive policy framework has also been recommended in the literature. Previous studies emphasized that additional measures could reduce the regressive nature of health taxes [39], limit the possibility for substitution to unhealthful alternatives [46, 47], or are necessary to achieve sustained reductions in SSB consumption [41]. For example, a tax combined with a subsidy for fruits and vegetables [39] and an extension of an SSB tax to a tax that taxes all caloric sweeteners could be more effective than a tax in isolation [47]. The WHO recommends comprehensive action plans at the country level that combine taxation, restriction of marketing of sugary products to children, and education [50]. Using revenue to fund initiatives benefiting people with greater disadvantage was recommended by a study conducted in Australia on modelled health benefits of an SSB tax across socioeconomic groups [42]. In a study exploring public acceptability of an SSB tax in the Netherlands, less than half of the Dutch adults (*n* = 500) were in favour of an SSB tax in general, but if the revenue is used for health initiatives more than half of the Dutch adults were in favour of an SSB tax [51]. Support for an SSB tax was significantly lower among people with a lower educational level than people with a higher educational level.

Furthermore, the World Cancer Research Fund International (WCRF) recommends modelling the impact of SSB taxes on different socioeconomic groups, to design an SSB tax that is fit-for-purpose and context appropriate, and to increase public and political support for the tax [52]. The Dutch Scientific Council for Government Policy proposes a perspective in which policymakers should not focus on decreasing health inequalities, but on where the greatest possible health gains lie, and how to keep health losses to a minimum [53]. This means a greater focus on people with the greatest health disadvantage.

### Strengths and limitations

The main strength of this study is that it is one of the first studies to provide insights into the views of various stakeholder groups (parliamentary parties, ministries, academia, health and consumer organizations, health professional associations, advisory bodies, trade associations) regarding the effects of an SSB tax on different socioeconomic groups. To ensure that all relevant stakeholder groups were included in our study, we asked each participant if stakeholders were lacking from the initial anonymized list of stakeholders developed by the research team.

Another strength of this study is the use of a qualitative design, as the interviews provided in-depth and rich information that could not have been gained through quantitative methods, or a systematic review of the available evidence of the effectiveness of an SSB tax on SSB consumption or health outcomes of different socioeconomic groups [54]. Insights into the stakeholder views via in-depth interviews lead to a greater understanding on how different stakeholders believe an SSB tax could contribute to socioeconomic inequalities in dietary intake and health and address arguments which may hinder the decision to implement an SSB tax. Furthermore, these perspectives provide useful suggestions how to increase the pro-equity effect of an SSB tax.

Our study also has some limitations. Firstly a considerable number of potential participants did not respond to our interview invitation (*n* = 8) or declined (*n* = 10), despite the reminder e-mails and calls made by the researchers. In particular, policymakers and politicians from whom we would expect opposition to the implementation of an SSB tax, stated that they did not want to participate or were too busy. This might be related to the politically sensitive nature of an SSB tax. A second limitation could be that the interviews were held by two different researchers (ME and SD). ME conducted interviews with stakeholders from academia, advisory bodies, trade associations, health professional associations, and health and consumer organizations. SD conducted interviews with stakeholders from parliamentary parties, ministries, and trade associations. We attempted to prevent interviewer bias by using the same interview guide and by close communication between the researchers during the data collection. Also, both researchers were trained in qualitative research. Lastly, it should be kept in mind that the findings of this study were restricted to the opinions of 27 stakeholders in the Netherlands and a substantial number of invited stakeholders (*n* = 19) declined to participate. Therefore, we are not certain if all opinions have been included in this study.

## Conclusions

Participants, from all stakeholder groups, mentioned that an SSB tax would have a larger impact on the budgets of people with a lower SEP. As a result, lower socioeconomic groups who often have unhealthier diets (including a higher SSB consumption) and are more likely to be overweight and obese, may be more likely of cutting down on SSBs. Therefore, participants, from all stakeholder groups except trade associations, indicated an SSB tax could have greater health benefits among lower socioeconomic groups than higher socioeconomic groups and has the potential to be pro-equity. However, some participants (from academia, a health/consumer organization, and a health professional association) did not agree on this pro-equity effect, as they believed that an SSB tax could also have no or adverse health effects (e.g. compensation with other unhealthy behaviour). These counterarguments may hinder the decision-making process to implement an SSB tax. To be effective and to prevent potential adverse health effects, some participants (from academia and a health/consumer organization) stressed the importance of additional interventions facilitating the reduction of SSB consumption in lower socioeconomic groups. These interventions would support the appropriate, effective and equitable development and implementation of an SSB tax. These interventions could also increase support for an SSB tax from several stakeholder groups [51, 52] and with that facilitate the decision-making process.

Further research is required to gain more insights into the opinions of certain stakeholder groups regarding the effects of an SSB tax on different socioeconomic groups. It would also be interesting to have more studies that statistically test differences in outcomes following an SSB tax between different socioeconomic groups, to substantiate the arguments and generate more evidence.

## Data Availability

The transcripts generated and analyzed during the current study are not publicly available due to ethical restrictions protecting participant confidentiality.

## Abbreviations

SEP: Socioeconomic position
SSB: Sugar-sweetened beverages
WCRF: World Cancer Research Fund International
WHO: World Health Organization
